# Systemic inflammation and its relationship with pruritus in early‐stage mycosis fungoides

**DOI:** 10.1111/jcmm.18125

**Published:** 2024-02-08

**Authors:** Berna Solak, Rabia Öztaş Kara

**Affiliations:** ^1^ Department of Dermatology, School of Medicine Sakarya University Sakarya Turkey

**Keywords:** itching, monocyte‐lymphocyte ratio, mycosis fungoides, neutrophil‐lymphocyte ratio, platelet‐lymphocyte ratio, pruritus, systemic immune‐inflammation index

## Abstract

The underlying mechanisms mycosis fungoides (MF)‐related pruritus remain unclear, and the link between pruritus and systemic inflammation in MF is unexplored. We aimed to investigate systemic inflammation in MF patients and its potential connection to pruritus. In this retrospective study, demographic characteristics, MF stage, clinical and laboratory findings, and neutrophil‐lymphocyte ratio (NLR), platelet‐lymphocyte ratio (PLR), monocyte‐lymphocyte ratio (MLR), systemic immune‐inflammation index (SII), systemic inflammation response index (SIRI) and pan‐immune inflammation value (PIV) were assessed for all participants. Additionally, mSWAT scores, Dermatology Life Quality Index (DLQI), and pruritus presence and intensity via Visual Analogue Scale (VAS) scoring were recorded for MF patients. A total of 81 patients with early‐stage MF and 50 controls were enrolled. Itching was present in 41 patients (50.6%). NLR, PLR, SII, SIRI and CRP values in the MF group were significantly higher. CRP, NLR, mSWAT and DLQI score were significantly higher in MF patients with pruritus than those without. Pruritus was positively correlated with DLQI, mSWAT, CRP, NLR, MLR and SIRI. VAS score was positively correlated with eosinophil count and DLQI. In the multivariate logistic regression model, only NLR was an independent and significant associate of pruritus in patients with MF. This study provides evidence of enhanced systemic inflammation in early‐stage MF patients. Additionally, the correlation between pruritus with mSWAT scores and systemic inflammation parameters suggests a potential link between pruritus and the inflammatory milieu in MF.

## 1 INTRODUCTION

Mycosis fungoides (MF) is the most common type of cutaneous T‐cell lymphoma (CTCL), which is a group of lymphoproliferative disorders characterized by the accumulation of malignant T cells in the chronically inflamed skin. While being localized to the skin and quite benign in the early stages, it is characterized by metastases and low survival in advanced stages. MF lesions, which carry a significant inflammatory burden, exhibit a predominant Th1 inflammatory microenvironment in the early stages, transitioning to a Th2‐dominated environment in the advanced stages.^1^ Although several inflammatory markers have been shown to play a role in pathogenesis, there is a noticeable scarcity of literature, particularly examining systemic inflammation in MF patients.

Pruritus is the most common and distressing symptom observed in MF patients.^2^ In MF, pruritus differs by showing a limited response to antihistamines and being more prevalent in MF types with a grim prognosis.^3^, ^4^ Furthermore, the mechanism underlying pruritus remains elusive. Hence, our study aimed to investigate the systemic inflammation profile in patients diagnosed with MF and determine any potential correlation with the occurrence of pruritus. To the best of our knowledge, to date, there is no study examining the relationship between pruritus and systemic inflammation in MF patients in the literature.

## 2 MATERIALS AND METHODS

This is a retrospective case–control study that encompassed MF patients under observation at the dermatology outpatient clinic of a tertiary care hospital from January 2011 to January 2023. Ethical approval was taken from the Local Ethics Committee (document number: E‐71522473‐050.01.04‐230,888‐91). Demographic characteristics, MF stage and other clinical and laboratory findings of the participants were reviewed.

The stage of MF patients was determined using established criteria, and severity was assessed by the modified Severity‐Weighted Assessment Tool (mSWAT) scores.^5^ Furthermore, mSWAT scores, the Dermatology Life Quality Index (DLQI), and the presence and intensity of itching were documented for MF patients.

Patients with incomplete medical records were excluded from the analysis. The control group comprised healthy volunteers without pruritus, matched for age and gender. Individuals with active infections, malignancies, chronic hepatic or renal diseases, and haematological and severe cardiovascular disorders were not included in the study.

The intensity of itching was quantified using Visual Analog Scale (VAS) scores. Systemic inflammation parameters, including the neutrophil‐lymphocyte ratio (NLR), platelet‐lymphocyte ratio (PLR), monocyte‐lymphocyte ratio (MLR), systemic immune‐inflammation index (SII), systemic inflammation response index (SIRI) and pan‐immune inflammation value (PIV) were evaluated for all participants. The SII and SIRI were calculated as follows: SII = platelet count × neutrophil count/lymphocyte count and SIRI = neutrophil count × monocyte count/lymphocyte count, respectively.^6^, ^7^ The PIV was calculated as follows: (neutrophil count × platelet count × monocyte count)/lymphocyte count.^8^

### 2.1 Statistical analysis

Statistical analysis was performed using the SPSS 25.0 statistical software package (SPSS Inc. Chicago, IL, USA). A probability value of *p* < 0.05 was considered statistically significant, and two‐tailed *p*‐values were reported to indicate the level of statistical significance for each comparison. The Kolmogorov–Smirnov test was used to check whether the distribution of the variables were normal. Continuous variables were presented as mean ± standard deviation or median (min–max) according to the normally distributed. Categorical variables were presented as numbers and percentages (%). The chi‐squared test was used to compare categorical variables between groups. The independent samples *t*‐test, or The Mann–Whitney *U*‐test, was used to compare continuous variables between two groups according to the distribution. The Kruskal–Wallis test was used to compare continuous variables between groups of more than two. Correlation between variables were evaluated using the Spearman's and the point biserial correlation analysis. To identify independent variables for pruritus in MF patients, multivariate logistic regression analysis was conducted. Variables with a *p*‐value <0.2, when compared between patients with and without pruritus (Table 4), were assessed in the univariate analysis. Significant variables identified in the univariate analysis were included in the multivariate model. Due to the high correlation between SIRI and NLR, only NLR was included in the model. Despite the mSWAT score having a *p*‐value <0.05 in the multivariate logistic regression analysis, it was not considered statistically significant due to the upper value of the 95% CI being 1.000.

## 3 RESULTS

### 3.1 Patient characteristics

A total of 81 patients with early‐stage MF and 50 age‐ and gender‐matched healthy controls were included in the study. The mean age of the patients was 48 ± 13.5 years, and 48.1% were women. Pruritus was observed in 41 patients (50.6%). The clinical characteristics and treatments of MF patients are presented in Table 1.

**TABLE 1:** Clinical findings, treatments and LDH results of the MF patients.

	MF Patients (*n* = 81)
Duration of MF (months)	41.0 (2.0–276.0)
mSWAT score	20.0 (1.0–110.0)
Presence of pruritus	41 (50.6%)
VAS score	6.0 (2.0–10.0)
TNM stage	
IA	32 (39.5%)
IB	32 (39.5%)
IIA	17 (21.0%)
Treatments	
Absent	27 (33.3%)
Topical steroids	31 (38.3%)
Topical retinoids	5 (6.2%)
NbUVB	2 (2.5%)
Interferon	1 (1.2%)
Systemic retinoic acid	7 (8.6%)
Topical steroids + topical retinoids	3 (3.7%)
Systemic retinoic acid + topical steroids	3 (3.7%)
NbUVB + topical steroids	1 (1.2%)
Systemic retinoic acid + topical retinoids	1 (1.2%)
Moisturizer use	27 (33.3%)
DLQI score	4.0 (0–22.0)
LDH (U/L)	197 ± 48.2

Abbreviations: DLQI, Dermatology Life Quality Index; LDH, lactate dehydrogenase; MF, mycosis fungoides; NbUVB, narrow band UVB phototherapy; LDH, lactate dehydrogenase; VAS, Visual Analogue Scale.

### 3.2 Inflammatory parameters

Notably, the MF group exhibited significantly elevated NLR, PLR, SII, SIRI and CRP values (*p* = 0.003, *p* < 0.001, *p* = 0.003, *p* = 0.041 and *p* = 0.004, respectively) and a significantly lower lymphocyte count compared to the control group (*p* = 0.009) (Table 2).

**TABLE 2:** Comparison of demographic characteristics, laboratory values, and inflammatory parameters between mycosis fungoides patients and the control group.

	Mycosis fungoides (*n* = 81)	Controls (*n* = 50)	*p*‐value
Sex			
Female	39 (48.1%)	26 (52.0%)	0.804
Male	42 (51.9%)	24 (48.0%)	
Age (years)	48 ± 13.5	47.2 ± 10	0.717
Uric acid (mg/dL)	5.2 ± 1.5	4.9 ± 1.2	0.190
Haemoglobin (g/dL)	13.9 ± 1.5	14.3 ± 1.5	0.178
White blood cell count (10^3^/μL)	7.20 ± 1.57	7.19 ± 1.50	0.973
Neutrophil count (10^3^/μL)	4.26 ± 1.22	4.05 ± 1.02	0.293
Lymphocyte count (10^3^/μL)	2.13 ± 0.56	2.43 ± 0.66	0.009
Monocyte count (10^3^/μL)	0.51 ± 0.16	0.52 ± 0.13	0.799
Eosinophil count (10^3^/μL)	0.10 (0.02–1.00)	0.15 (0.03–0.47)	0.198
Platelet count (10^3^/μL)	253.3 ± 63.2	240.8 ± 41.6	0.177
C‐reactive protein (mg/L)	3.8 ± 2.3	2.9 ± 0.9	0.004
NLR	1.92 (1.08–5.54)	1.66 (0.98–2.89)	0.003
MLR	0.24 (0.03–0.65)	0.22 (0.12–0.52)	0.124
PLR	124.1 ± 37.4	103.3 ± 22.4	<0.001
SII	478.5 (214.3–1611.7)	382.0 (204.1–770.7)	0.003
SIRI	0.98 (0.17–3.88)	0.81 (0.32–2.54)	0.041
PIV	278.4 (58.4–1128.2)	192.9 (61.2–614.8)	0.063

Abbreviations: DLQI, Dermatology Life Quality Index; LDH, lactate dehydrogenase; MLR, monocyte‐lymphocyte ratio; mSWAT, modified Severity‐Weighted Assessment Tool; NLR, neutrophil‐lymphocyte ratio; PIV, pan‐immune inflammation value; PLR, platelet‐lymphocyte ratio; SII, systemic immune‐inflammation index; SIRI, systemic inflammation response index.

### 3.3 Stage‐related pruritus characteristics and inflammation

**FIGURE 1:**
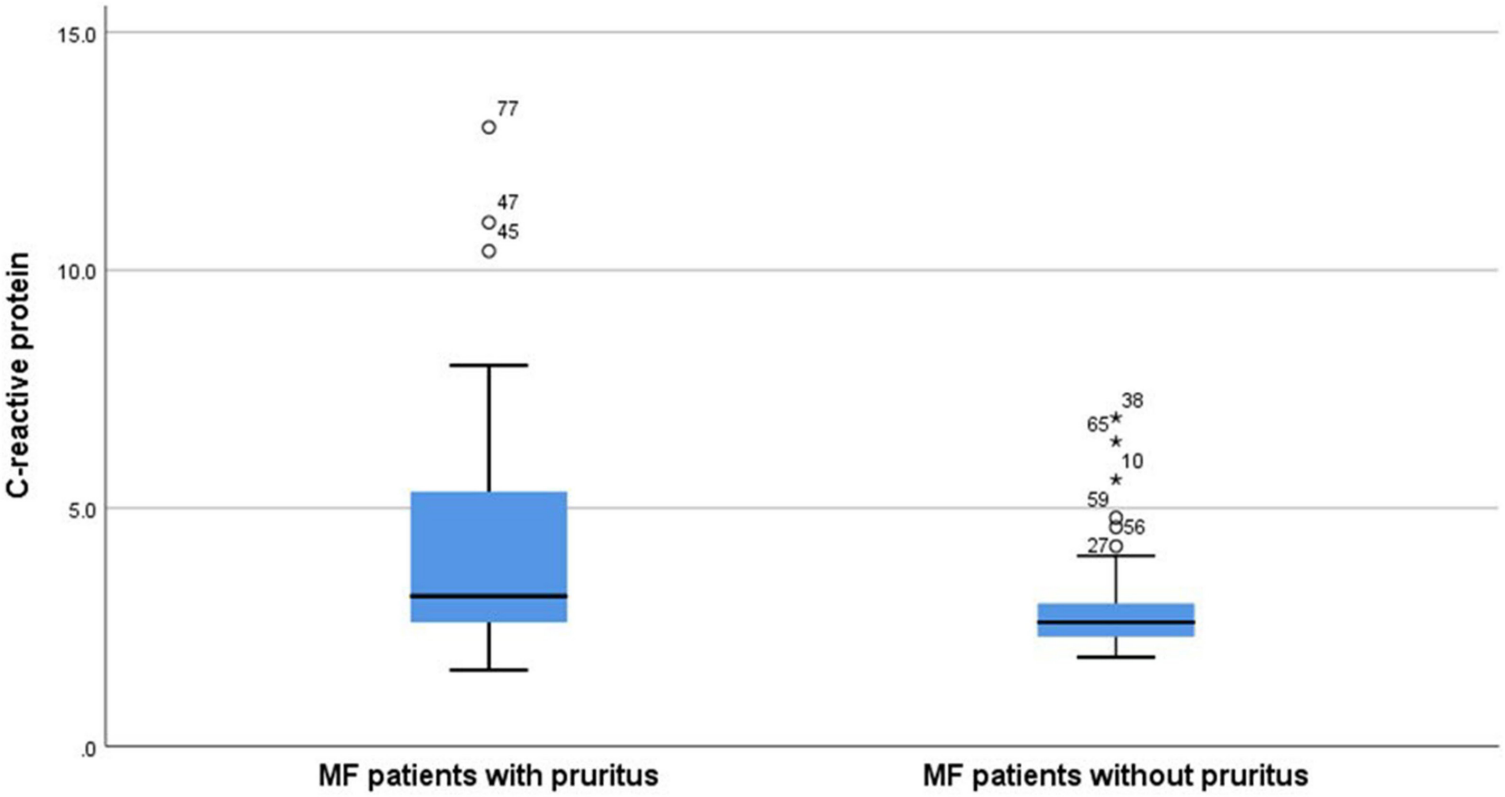
Box plot graph illustrates the C‐reactive protein (CRP) levels in mycosis fungoides (MF) patients with and without pruritus.

**FIGURE 2:**
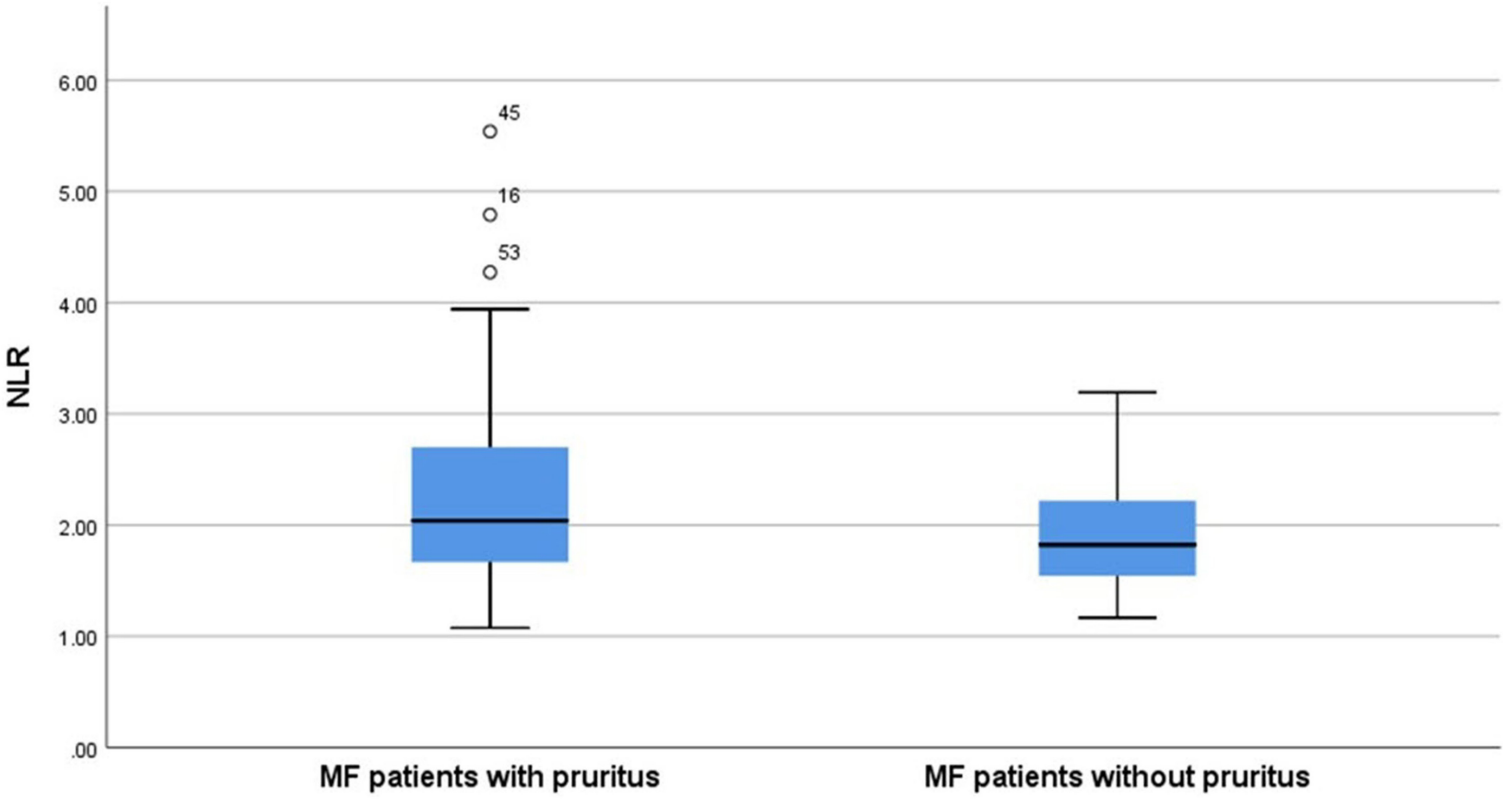
Box plot graph displays the neutrophil‐lymphocyte ratio (NLR) values in mycosis fungoides (MF) patients with and without pruritus.

According to disease stages, the comparison of pruritus characteristics, laboratory values and inflammatory parameters among MF patients are illustrated in Table 3. In MF patients, those with pruritus displayed significantly higher CRP (Figure 1), NLR (Figure 2), mSWAT and DLQI scores compared to those without pruritus (*p* = 0.031, *p* = 0.048, *p* = 0.040 and *p* < 0.001, respectively) (Table 4).

**TABLE 3:** Comparison of mycosis fungoides patients based on stages in terms of pruritus features, laboratory values and inflammatory parameters.

	Stage IA (*n* = 32)	Stage IIA (*n* = 32)	Stage IIB (*n* = 17)	*p*‐value
Presence of pruritus	13 (40.6%)	19 (59.4%)	9 (52.9%)	0.317
VAS score	5.0 (3.0–10.0)	6.0 (2.0–10.0)	7.0 (4.0–10.0)	0.763
LDH (U/L)	200 (119–330)	176 (121–283)	186 (161–389)	0.285
WBC count (10^3^/μL)	7.20 (4.10–11.40)	6.85 (4.90–10.15)	7.60 (4.90–11.90)	0.565
Neutrophil count (10^3^/μL)	4.18 (2.20–7.20)	4.00 (2.60–6.74)	4.30 (2.80–9.10)	0.975
Lymphocyte count (10^3^/μL)	2.10 (1.30–3.90)	2.00 (1.10–2.70)	2.19 (1.40–3.90)	0.144
Monocyte count (10^3^/μL)	0.50 (0.20–0.86)	0.51 (0.23–0.90)	0.48 (0.10–0.90)	0.810
Eosinophil count (10^3^/μL)	0.16 (0.03–0.60)	0.10 (0.03–1.00)	0.20 (0.02–0.40)	0.245
Platelet count (10^3^/μL)	235.5 (161–424)	242 (133–384)	277 (148–450)	0.198
C‐reactive protein (mg/L)	2.7 (1.6–13.0)	2.8 (2.1–6.9)	4.1 (1.9–7.5)	0.486
NLR	1.93 (1.08–5.54)	2.10 (1.24–4.27)	1.73 (1.23–4.79)	0.153
MLR	0.22 (0.10–0.54)	0.26 (0.14–0.65)	0.21 (0.03–0.40)	0.277
PLR	117.9 (65.1–249.4)	118.8 (63.2–195.5)	112.3 (81.6–187.5)	0.586
SII	447.2 (245.3–1611.7)	526.1 (214.3–1039.4)	458.1 (272.9–1029.7)	0.615
SIRI	0.92 (0.32–3.88)	1.07 (0.42–3.08)	0.84 (0.17–2.87)	0.296
PIV	219.6 (58.9–1128.2)	276.2 (89.1–661.4)	226.9 (58.4–617.8)	0.655

Abbreviations: LDH, lactate dehydrogenase; MLR, monocyte‐lymphocyte ratio; NLR, neutrophil‐lymphocyte ratio; PIV, pan‐immune inflammation value; PLR, platelet‐lymphocyte ratio; SII, systemic immune‐inflammation index; SIRI, systemic inflammation response index; VAS, Visual Analogue Scale; WBC, white blood cell.

**TABLE 4:** Comparison of mycosis fungoides patients with and without pruritus in relation to demographic characteristics, laboratory values and inflammatory parameters.

	MF with pruritus (*n* = 41)	MF without pruritus (*n* = 40)	*p*‐value
Sex			
Female	20 (48.8%)	19 (47.5%)	>0.999
Male	21 (51.2%)	21 (52.5%)	
Age (years)	49.6 ± 13.9	46.3 ± 13.1	0.272
Duration of MF (months)	36.0 (7.0–240.0)	49.0 (2.0–276.0)	0.509
mSWAT score	24.0 (3.0–110.0)	13.0 (1.0–80.0)	0.040
Types of lesions			
Patch	17 (41.5%)	20 (50.0%)	0.441
Plaque	24 (58.5%)	20 (50.0%)	
TNM stage			
IA	13 (31.7%)	19 (47.5%)	0.317
IB	19 (46.3%)	13 (32.5%)	
IIA	9 (22.0%)	8 (20.0%)	
DLQI score	8.0 (1.0–22.0)	1.0 (0.0–8.0)	<0.001
Treatment for MF	26 (63.4%)	28 (70.0%)	0.694
Moisturizer use	13 (31.7%)	14 (35.0%)	0.937
LDH (U/L)	201.0 ± 47.2	192.5 ± 49.6	0.463
Uric acid (mg/dL)	5.2 ± 1.4	5.2 ± 1.6	0.905
Haemoglobin (g/dL)	13.7 ± 1.5	14.2 ± 1.5	0.095
White blood cell count (10^3^/μL)	7.37 ± 1.71	7.02 ± 1.43	0.315
Neutrophil count (10^3^/μL)	4.49 ± 1.44	4.02 ± 0.90	0.084
Lymphocyte count (10^3^/μL)	2.06 ± 0.55	2.21 ± 0.56	0.236
Monocyte count (10^3^/μL)	0.52 ± 0.15	0.50 ± 0.17	0.516
Eosinophil count (10^3^/μL)	0.14 (0.03–1.00)	0.10 (0.02–0.40)	0.175
Platelet count (10^3^/μL)	249.0 ± 54.0	257.6 ± 71.9	0.543
C‐reactive protein (mg/L)	4.4 ± 2.8	3.2 ± 1.3	0.031
NLR	2.04 (1.08–5.54)	1.82 (1.17–3.19)	0.048
MLR	0.26 (0.03–0.65)	0.22 (0.11–0.41)	0.077
PLR	126.8 ± 36.0	121.3 ± 39.0	0.509
SII	530.0 (229.1–1611.7)	449.7 (214.3–900.0)	0.126
SIRI	1.08 (0.17–3.88)	0.92 (0.32–1.72)	0.059
PIV	275.6 (58.4–1128.2)	213.8 (63.6–541.2)	0.093

Abbreviations: DLQI, Dermatology Life Quality Index; LDH, lactate dehydrogenase; MLR, monocyte‐lymphocyte ratio; mSWAT, modified Severity‐Weighted Assessment Tool; MF, mycosis fungoides; NLR, neutrophil‐lymphocyte ratio; PIV, pan‐immune inflammation value; PLR, platelet‐lymphocyte ratio; SII, systemic immune‐inflammation index; SIRI, systemic inflammation response index.

### 3.4 Treatment impact on inflammatory parameters

There were no differences between patients receiving and not receiving treatment for MF in terms of demographic characteristics, duration of MF, mSWAT score, presence of pruritus, VAS score, TNM stage, DLQI score and treatment for MF laboratory values, and systemic inflammatory parameters.

### 3.5 Moisturizer usage and inflammatory parameters

When compared MF patients who used moisturizer regularly every day with those who do not, no statistically significant difference was observed in terms of demographic characteristics, duration of MF, mSWAT score, presence of pruritus, VAS score, TNM stage, DLQI score, treatment for MF laboratory values and systemic inflammatory parameters.

## 4 CORRELATIONS

**FIGURE 3:**
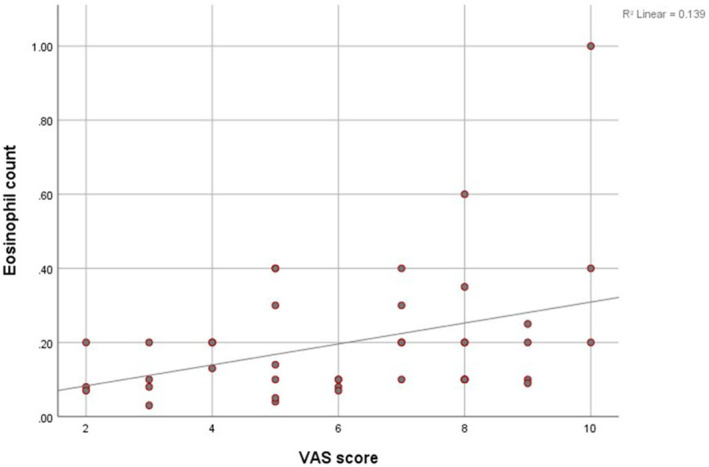
Scatter plot depicts the correlation between eosinophil count and Visual Analogue Scale (VAS) score in mycosis fungoides (MF) patients.

Pruritus exhibited correlations with DLQI, mSWAT, CRP, NLR, MLR and SIRI (*r* = 0.607, *p* < 0.001; *r* = 0.235, *p* = 0.035; *r* = 0.270, *p* = 0.036; *r* = 0.276, *p* = 0.013; *r* = 0.221, *p* = 0.047 and *r* = 0.267, *p* = 0.016, respectively). The VAS score showed positive correlations with eosinophil count (Figure 3) and DLQI (*r* = 0.373, *p* = 0.016 and *r* = 0.426, *p* = 0.006, respectively). The eosinophil count was correlated with NLR, MLR and SIRI (*r* = 0.228, *p* = 0.040; *r* = 0.320, *p* = 0.004 and *r* = 0.369, *p* = 0.001, respectively).

## 5 LOGISTIC REGRESSION ANALYSIS

Logistic regression analysis for the presence of pruritus in MF patients is presented in Table 5. In the multivariate logistic regression model, only NLR was an independent and significant associate of pruritus in patients with MF.

**TABLE 5:** Binary logistic regression analysis to determine independent associates of presence of pruritus in MF patients.

Variables	Univariate LR			Multivariate LR		
	OR	95% CI	*p*‐value	OR	95% CI	*p*‐value
mSWAT score	0.977	0.955–0.999	0.044	0.976	0.967–1.000	0.049
Haemoglobin	1.290	0.955–1.741	0.097			
Neutrophil	0.714	0.482–1.056	0.092			
C‐reactive protein	0.740	0.544–1.005	0.054			
NLR	0.413	0.190.878	0.022	0.364	0.140–0.944	0.024
MLR	0.011	0.000–1.101	0.055			
SII	0.998	0.996–1.000	0.083			
SIRI	0.338	0.129–0.882	0.027			
PIV	0.997	0.994–1.000	0.072			

*Note*: Hosmer and Lemeshow test *p* = 0.068.

Abbreviations: MF, mycosis fungoides; MLR, monocyte‐lymphocyte ratio; mSWAT, modified Severity‐Weighted Assessment Tool; NLR, neutrophil‐lymphocyte ratio; PIV, pan‐immune inflammation value; SII, systemic immune‐inflammation index; SIRI, systemic inflammation response index.

## 6 DISCUSSION

The significant findings of the current study are as follows: (i) MF patients had significantly higher NLR, PLR, SII, SIRI and CRP values, coupled with a lower lymphocyte count compared to controls. (ii) Among MF patients, those with pruritus showed significantly elevated levels of CRP, NLR, mSWAT and DLQI scores compared to those without pruritus. (iii) Pruritus was significantly correlated with DLQI, mSWAT, CRP, NLR, MLR and SIRI. (iv) The VAS score correlated positively solely with eosinophil count and DLQI score. (v) NLR appeared as the sole independent predictor of pruritus in the logistic regression analysis.

Limited studies exist on systemic inflammatory markers in MF patients. Addressing this gap, our study explores the relationship between these markers and MF clinical manifestations, offering insights into potential prognostic and diagnostic implications. In a study comparing MF patients with healthy controls, high‐sensitivity CRP was found to be significantly elevated in MF patients compared to controls.^9^ This observation aligns with our findings, suggesting the presence of chronic inflammation in MF. In a study investigating the prognostic significance of CRP levels in patients with MF and Sézary syndrome, it was demonstrated that elevated CRP levels were associated with advanced stages and lower treatment response rates. The authors postulated that CRP could serve as a valuable prognostic factor for risk assessment and treatment decisions, attributing the link between CRP and disease progression to inflammation within the tumour microenvironment.^10^ In our study, we also found CRP to be significantly elevated in MF patients compared to the control group. However, we did not observe any differences in CRP levels among stages (Stage IA, IB and IIA).

In a meta‐analysis on peripheral T‐cell lymphomas (PTCL), high NLR correlated with worse overall survival in PTCL patients (921 patients, 8 studies), highlighting NLR's prognostic potential in PTCL.^11^ Cosimo Di Raimondo and colleagues^12^ observed in 302 MF patients that advanced‐stage MF correlated with higher NLR (median 2.64), while early‐stage patients had lower NLR (median 1.88), suggesting NLR's role in identifying advanced stages with 67.6% sensitivity and 60.4% specificity. Cengiz et al.^13^ found that high NLR values at the time of diagnosis were associated with an advanced disease stage. Additionally, they observed elevated NLR values in patients with a high beta‐2‐microglobulin concentration, suggesting the potential of NLR to identify high‐risk patients. Based on the ROC curve analysis, the authors identified a potential threshold value (NLR > 2.85) for identifying patients at high risk for advanced disease stage and disease progression.^13^ In the study conducted by Eren et al.,^14^ set the NLR cut‐off at two, finding no significant differences in treatment demand or progression based on NLR (in 117 MF patients).^14^ In our study, MF patients showed differences in CRP, NLR, PLR, SII and SIRI compared to controls, with no significant stage‐based variations, suggesting a consistent inflammatory burden in early stages. However, in the multivariate logistic regression model, NLR was an independent and significant associate of pruritus in patients with MF. Conflicting findings across studies may stem from population variations.

In lymphomas, low lymphocyte count is associated with poor prognosis.^15^, ^16^ In a study carried out by Abeni and colleagues on MF patients, low CD8+ lymphocyte count (<600/mL), high WBC, and neutrophil count were found to be significantly associated with lower survival probability.^17^ However, conflicting findings exist. Another study conducted on advanced stages of MF and Sézary syndrome demonstrated that low absolute lymphocyte count is not a prognostic factor for overall survival. In our study, we observed a significantly higher NLR value in MF patients compared to the control group. This difference appears to be driven by decreased lymphocyte levels rather than elevated neutrophils, as lymphocytes were significantly lower in the MF group, while neutrophils showed no significant difference. The decline in lymphocytes, as the denominator of NLR, results in an increase in this ratio.

Pruritus is a frequently overlooked but impactful symptom in MF patients, affecting approximately 50% of individuals.^18^ This symptom adversely affects sleep, daily activities and can lead to public embarrassment due to scratching.^3^, ^4^ In our study, we found the presence of pruritus in MF patients to be at a rate of 50.6%, and we observed a significantly higher DLQI score in MF patients with pruritus compared to those without pruritus. The pathogenesis of pruritus in MF remains enigmatic, as it often does not respond adequately to antihistamines, hinting at a non‐histamine pathway.^19^, ^20^

Much like the case of chronic atopic dermatitis, eosinophils may also play a significant role in causing inflammation and itching in MF.^21^ In another study, it was demonstrated that elevated blood eosinophil levels were an independent factor contributing to the progression of CTCLs and disease‐specific mortality.^22^ While our study found no significant difference in eosinophil counts between MF patients and the control group or across MF stages, there was a positive correlation between Visual Analog Scale (VAS) scores and eosinophil counts. Additionally, we noted associations between eosinophil counts and inflammation markers (NLR, MLR and SIRI).

In a study, disease severity scores on the mSWAT scale were significantly higher in MF patients who experienced itching, indicating a more severe condition.^23^ In our study, the mSWAT score was significantly higher in MS patients with pruritus compared to those without pruritus. Furthermore, pruritus demonstrated a correlation with the mSWAT score. However, we did not find the mSWAT score to be an independent variable for itching in the multivariate regression analysis.

When examining the relationship between inflammatory markers and itching in MF patients, there is currently no existing study in the literature investigating the association between pruritus and systemic inflammation markers in MF patients. In our study, we observed that CRP and NLR were significantly higher in MF patients with pruritus compared to those without pruritus. Additionally, pruritus exhibited a mild correlation with CRP, NLR, MLR and SIRI. These data imply that in the pathogenesis of itching in MF patients, whether accompanied by eosinophils or not, a systemic inflammation could potentially play a role.

SII is the combination of platelets with NLR, while SIRI is the addition of monocytes to NLR. The absence of a difference in PLR, SII and SIRI, despite a difference in NLR between MF patients with and without pruritus, suggests that in pruritus of MF patients, neutrophils or lymphocytes might play a more predominant role rather than monocytes or platelets, or that the mechanism of pruritus might occur through a different pathway. Consequently, this could potentially influence NLR as a secondary factor. However, the limited sample size might have hindered the statistical significance of the difference from being achieved.

The main limitation of our retrospective study includes its design and a small patient cohort, potentially hindering statistical significance in subgroup comparisons for this rare disease. As a third limitation, it can be stated that being a single‐centre study might have introduced confounding factors, such as environmental variables like seasonal allergens and cohort‐specific conditions, which could affect pruritus and systemic inflammatory markers. Despite all of these limitations, we believe that our study's results are valuable due to its comprehensive examination of numerous indicators in MF patients and being the first investigation into the role of systemic inflammation in the aetiology of pruritus in this context.

In conclusion, this study has shed light on the enhanced systemic inflammation in early‐stage MF patients. Furthermore, the correlation between pruritus with mSWAT scores and systemic inflammation parameters suggests a plausible association between pruritus and the inflammatory milieu in MF. We believe that investigating inflammation remains an essential avenue to comprehending the pathogenesis of MF patients and illuminating the aetiology of pruritus, a distressing symptom that frequently resists treatment.

## AUTHOR CONTRIBUTIONS

**Berna Solak:** Conceptualization (lead); formal analysis (lead); investigation (lead); methodology (lead); resources (equal); supervision (equal); writing – original draft (lead); writing – review and editing (lead). **Rabia Öztaş Kara:** Conceptualization (supporting); investigation (supporting); resources (equal); supervision (equal); writing – original draft (supporting); writing – review and editing (supporting).

## FUNDING INFORMATION

There are no funding sources to declare.

## CONFLICT OF INTEREST STATEMENT

No conflict of interest.

## Data Availability

The data that support the findings of this study are available from the corresponding author, upon reasonable request.
